# Hypertension in Pregnancy in the US—One Step Closer to Better Ascertainment and Management

**DOI:** 10.1001/jamanetworkopen.2020.19364

**Published:** 2020-10-01

**Authors:** Elena V. Kuklina

**Affiliations:** Division of Reproductive Health, National Center for Chronic Disease Prevention and Health Promotion, US Centers for Disease Control and Prevention, Atlanta, Georgia.

The study by Butwick et al^1^ examined the prevalence of prepregnancy (chronic) and pregnancy-associated hypertension using 2017 US birth certificate data. The 2003 version of the birth certificate that was in use in all states in 2017 provides checkboxes for prepregnancy (chronic) hypertension, gestational hypertension, and eclampsia.^2^ Gestational hypertension includes transient hypertension, preeclampsia, and hemolysis, elevated liver enzyme levels, and low platelet levels syndrome.^2^ Thus, while Butwick et al^1^ use the term *hypertensive disorders of pregnancy, gestational hypertension* is used as an overarching term and applied to any hypertensive disorder diagnosed after 20 weeks of pregnancy. A recorder of a birth certificate can select eclampsia with either chronic hypertension or gestational hypertension. The authors report the prevalence of chronic hypertension (1.9%), gestational hypertension (6.5%), and eclampsia (0.3%) in the US, with the overall prevalence of any hypertension being 8.6% during pregnancy.^1^ Using the 2017 data from more than 3 500 000 births in analyses adjusted for patient-level factors, prevalence estimates of chronic hypertension were the lowest in Hawaii (1.0%; 95% CI, 0.9%-1.2%) and the highest in Alaska (3.4%; 95% CI, 3.0%-3.9%). The prevalence estimates of gestational hypertension were lowest in Massachusetts (4.3%; 95% CI, 4.1%-4.6%) and highest in Louisiana (9.3%; 95% CI, 8.9%-9.8%). The reported adjusted prevalence of eclampsia among states ranged from 0.03% in Delaware (95% CI, 0.01%-0.09%) to 2.8% in Hawaii (95% CI, 2.2%-3.4%) or 3 to 280 per 10 000 births. In addition to Hawaii, 3 other states (Alabama, Alaska, and Virginia) had rates of eclampsia greater than 1% or 100 per 10 000 births. The median difference in the adjusted odds ratio (MOR) assessed the variation in estimated odds of outcomes between states. The MOR varied by outcomes with a substantially greater MOR observed for eclampsia (MOR, 2.36; 95% CI, 1.88-2.82) than for chronic hypertension (MOR, 1.27; 95% CI, 1.20-1.33) or gestational hypertension (MOR, 1.17; 95% CI, 1.17-1.21).

Vital statistics records, such as birth certificates, provide a relatively quick and inexpensive way to monitor select disease conditions over time and identify unusual patterns of prevalence using large population-based data.^2^ The high degree of variation, as measured by the median difference in the adjusted odds ratio, in the prevalence of eclampsia among states and relatively high prevalence of eclampsia (>1% or 100 per 10 000 live births) in a few states, as reported by Butwick et al,^1^ calls for a more in-depth investigation of these differences. The authors attributed the observed discordance in the prevalence of gestational hypertension and chronic hypertension with eclampsia among several states to possible underreporting or underdiagnosis of gestational and chronic hypertension. Recording practices or underdiagnosis can affect state differences in the prevalence of eclampsia. The authors also mentioned other factors, including differences in clinical management, state-level economic factors, quality and access to antenatal and intrapartum care, and environmental etiologic factors. Clinical management practices are the most modifiable factors influencing the prevalence of eclampsia independent of its precursors' prevalence.^3^

Eclampsia is well accepted as both an indicator of severe maternal morbidity and a preventable condition.^3^ According to the American College of Obstetricians and Gynecologists, diagnosis of pregnancy-associated hypertension usually occurs after 20 weeks of gestation, with hypertension in the obstetric population defined as systolic blood pressure 140 mm Hg or greater or diastolic blood pressure 90 mm Hg or greater.^3^ Pregnancy-associated hypertension includes gestational hypertension (hypertension alone), preeclampsia (hypertension plus proteinuria or multiorgan dysfunction), and eclampsia (seizures). It may be debatable whether gestational hypertension, preeclampsia, or eclampsia are a continuum of 1 disease. Clinical progression from gestational hypertension to preeclampsia or preeclampsia to eclampsia can happen in a short time, sometimes in minutes, and each may develop without symptoms of the other conditions. The standard intervention for eclampsia prevention is the prompt administration of intravenous blood pressure medication and magnesium sulfate for persistent severe hypertension. As highlighted in the American College of Obstetricians and Gynecologists Practice Bulletin, “Gestational Hypertension and Preeclampsia,”^3^ treatment with magnesium sulfate for prevention and treatment of seizures should be initiated for severe hypertension regardless of the hypertension diagnosis, ie, gestational hypertension, preeclampsia, or eclampsia. Prompt, evidence-based management of severe hypertension (systolic blood pressure ≥160 mm Hg or diastolic blood pressure ≥110 mm Hg) is essential for the prevention of congestive heart failure, myocardial ischemia, ischemic stroke, or hemorrhagic stroke among women with any hypertensive disorders. Several countries have nationwide registries of severe maternal morbidities, including eclampsia, that allow examination of the effects of clinical practice on prevalence. For instance, the results from national registration studies of preeclampsia and eclampsia in the Netherlands (2004-2006) and the UK (2005-2006) showed a higher incidence of eclampsia in the Netherlands (5.4 per 10 000 deliveries) compared with the UK (2.7 per 10 000 deliveries).^4^ While the prevalence of preeclampsia with use of magnesium sulfate prophylaxis was similar between the countries, the Netherlands reported significantly lower use of antihypertensive medication (16% vs 71%) and higher thresholds for treatment of hypertension compared with the UK. After a decade of efforts to decrease the incidence of eclampsia through mandatory professional training promoting the use of antihypertensive medications for prevention of eclampsia, the incidence of eclampsia declined to 1.8 per 10 000 births in 2013-2016 in the Netherlands.^5^

Monitoring the prevalence of eclampsia and the influence of current clinical practice on hypertension management is challenging in the US on a national level owing to gaps in data availability and standardization. A 2015 to 2016 quality improvement project that standardized data collection among 23 community hospitals in the US demonstrated that consistent follow-up using state and national treatment guidelines resulted in a significant reduction in eclampsia rate (from 11.5 to 6.2 per 10 000 deliveries).^6^ This reduction was achieved in a relatively short period (18 months) with a high level of adherence. In addition to the timely administration of intravenous blood pressure medication (hydralazine or labetalol) and magnesium sulfate (30-60 minutes after detection of persistent severe hypertension), the intervention also included postpartum follow-up (7-14 days after delivery). The authors noted that monitoring of adherence was critical for successful implementation of the intervention. The Perinatal Quality Collaboratives are state networks of clinical services that offer another venue for rapid clinical quality improvement on the management of hypertensive disorders in pregnancy in the US. For example, the prevalence of severe complications among women with severe preeclampsia or eclampsia was reduced from 20% to 17.6% during an almost 1.5-year period among 13 hospitals participating in the Preeclampsia Collaborative in California.^7^ The building of a data center in 2013 to 2014 by housing a comprehensive data set with elements from birth certificates, patient hospital discharge records, and other clinical elements was a cornerstone of the initiative.^7^ This data set allowed comparing hospital performance with statistics at the county, system, regional, and statewide levels. Analysis at the patient and physician level provided opportunities to identify specific quality improvement approaches. The Illinois Perinatal Quality Collaboratives developed a multicomponent strategy using prompt treatment of severe hypertension (within ≤60 minutes), patient education, postpartum follow-up (7-10 days), and clinical debriefings using quality improvement processes. Improvements in each of these components were achieved after implementation. There was also a 27% decrease in the overall rate of severe maternal morbidity and pregnancy-related mortality during 2 years (2016-2017).

Thus, the study by Butwick et al^1^ is a valuable contribution to the current literature on hypertension during pregnancy in the US and variation by states. This analysis is also a useful step in untangling complex issues of ascertainment and management of hypertension during pregnancy with an ultimate goal of decreasing adverse outcomes.
